# Blocking autophagy overcomes resistance to dual histone deacetylase and proteasome inhibition in gynecologic cancer

**DOI:** 10.1038/s41419-022-04508-2

**Published:** 2022-01-17

**Authors:** Jianling Bi, Yuping Zhang, Paige K. Malmrose, Haley A. Losh, Andreea M. Newtson, Eric J. Devor, Kristina W. Thiel, Kimberly K. Leslie

**Affiliations:** 1 Department of Obstetrics and Gynecology, University of Iowa, Iowa City, IA 52242 USA; 2 Holden Comprehensive Cancer Center, University of Iowa, Iowa City, IA 52242 USA; 3 Division of Molecular Medicine, Departments of Internal Medicine and Obstetrics and Gynecology, the University of New Mexico Comprehensive Cancer Center, University of New Mexico Health Sciences Center, Albuquerque, NM 87131 USA

**Keywords:** Gynaecological cancer, Preclinical research

## Abstract

Histone deacetylase (HDAC) inhibitors and proteasome inhibitors have been approved by the FDA for the treatment of multiple myeloma and lymphoma, respectively, but have not achieved similar activity as single agents in solid tumors. Preclinical studies have demonstrated the activity of the combination of an HDAC inhibitor and a proteasome inhibitor in a variety of tumor models. However, the mechanisms underlying sensitivity and resistance to this combination are not well-understood. This study explores the role of autophagy in adaptive resistance to dual HDAC and proteasome inhibition. Studies focus on ovarian and endometrial gynecologic cancers, two diseases with high mortality and a need for novel treatment approaches. We found that nanomolar concentrations of the proteasome inhibitor ixazomib and HDAC inhibitor romidepsin synergistically induce cell death in the majority of gynecologic cancer cells and patient-derived organoid (PDO) models created using endometrial and ovarian patient tumor tissue. However, some models were not sensitive to this combination, and mechanistic studies implicated autophagy as the main mediator of cell survival in the context of dual HDAC and proteasome inhibition. Whereas the combination of ixazomib and romidepsin reduces autophagy in sensitive gynecologic cancer models, autophagy is induced following drug treatment of resistant cells. Pharmacologic or genetic inhibition of autophagy in resistant cells reverses drug resistance as evidenced by an enhanced anti-tumor response both in vitro and in vivo. Taken together, our findings demonstrate a role for autophagic-mediated cell survival in proteasome inhibitor and HDAC inhibitor-resistant gynecologic cancer cells. These data reveal a new approach to overcome drug resistance by inhibiting the autophagy pathway.

## Introduction

Endometrial and ovarian cancer are two of the most common gynecologic malignancies. These diseases combined lead to 25,000 deaths annually in the US [1]. Chemotherapy with the doublet of a platinum compound and a taxane is the first choice for most high-grade serous ovarian and high-risk endometrial cancers [2, 3]. While many patients have a good response to initial treatment, the majority develop recurrent disease and become resistant to chemotherapy. Thus, the development of effective therapies against gynecological cancers is still an unmet medical need.

The ubiquitin–proteasome system is responsible for the degradation of unnecessary or damaged proteins within mammalian cells [4]. Cancer cells rely heavily on hyperactivated proteasomes to evade programmed cell death [5]. Proteasome inhibition thereby prevents clearance of misfolded proteins and leads to cell death, which makes proteasome inhibitors promising therapeutic agents against cancer [6]. Currently, three proteasome inhibitors are approved by the FDA and in clinical use to treat multiple myeloma: bortezomib, carfilzomib, and ixazomib. Ixazomib was the first oral proteasome inhibitor to gain FDA approval [7]. Clinical trials have evaluated the efficacy and safety of bortezomib (NCT00023712) and carfilzomib (NCT00531284) in ovarian cancer [8]. Bortezomib in combination with conventional chemotherapy has also been tested in ovarian cancer (NCT00059618, NCT00028912, NCT01074411, NCT00620295, and NCT00667641) [8]. While these agents had a good safety and tolerability profile, no therapeutic effect was observed using either bortezomib or carfilzomib as a single agent or in combination with chemotherapy in ovarian cancer [9–11]. To date, analyses have not been performed to explain or overcome resistance to therapy found in these studies.

Histone deacetylases (HDACs) are a class of enzymes that epigenetically regulate many biological processes by deacetylating histones and other regulatory proteins [12]. Cancer cells maintain epigenetic modifications to regulate gene expression patterns that facilitate and sustain tumorigenesis [13]. HDAC inhibitors block the actions of HDACs and affect the expression of genes that regulate many cellular processes, including cell cycle, reactive oxygen species, and angiogenesis [14]. To date, four HDAC inhibitors have been FDA-approved for the treatment of lymphoma: vorinostat (SAHA), romidepsin, belinostat, and panobinostat. HDAC inhibitors employed alone or in combination with chemotherapy have been studied in ovarian and endometrial cancer clinical trials (NCT00772798, NCT00976183, NCT00993616, and NCT03018249), including our own group’s analysis of entinostat in a surgical window of opportunity trial in endometrial cancer [8, 15, 16]. Similar to proteasome inhibitors, HDAC inhibitors were well tolerated in ovarian cancer. Some studies reported limited efficacy as single agents [15], and a trial of vorinostat with chemotherapy was terminated due to toxicity. However, belinostat in combination with chemotherapy was well-tolerated with an encouraging 43% overall response rate [17].

Instead of using each agent individually or with chemotherapy, where success has been relatively modest, treatment with a proteasome inhibitor in combination with an HDAC inhibitor has achieved remarkable clinical success in hematological malignancies [4]. Numerous preclinical studies have also shown that a proteasome inhibitor and HDAC inhibitor combination can synergistically induce cell death in solid tumors [18–20]. Moreover, multiple clinical trials with proteasome and HDAC inhibitors are ongoing in solid tumors, some of which have shown promising results [21]. For example, the combination of vorinostat with the proteasome inhibitor marizomib was tested in a Phase I clinical trial (NCT00667082) of advanced or recurrent solid tumors. The data showed stable disease in 61% of evaluable patients [22]. Similarly, the combination of vorinostat with bortezomib in patients with advanced solid tumors (NCT00227513) resulted in stable disease in most patients [23]. However, this combination has not been yet tested in clinical trials of women with gynecologic malignancies.

While preclinical studies have demonstrated the activity of HDAC and proteasome inhibitors in solid tumors [24–26], including our own work in gynecologic cancer cell lines [27], the mechanisms underlying sensitivity vs. resistance to this combination are not well understood. Herein we demonstrate that gynecologic cancer cell lines, xenografted animal models, and patient-derived organoids (PDOs) of endometrial and ovarian cancer have differential sensitivity to the combination of the FDA-approved proteasome inhibitor ixazomib and the FDA-approved HDAC inhibitor romidepsin based upon their ability to induce autophagy as a survival mechanism. Thus, our data implicate autophagy as a major mediator of resistance, and inhibiting autophagy significantly enhances sensitivity to therapy. These studies set the stage for novel combinations to treat advanced and recurrent endometrial and ovarian cancer.

## Materials and methods

A full description of the Materials and methods are included in Supplemental Methods.

### PDO models

All studies using human tissues were approved by the University of Iowa (UI) Institutional Review Board (IRB), protocol #201809807. PDO cultures were created as we previously described [28].

### Western blotting

Western blotting was performed in lysates from cells or tumor tissues [29]. Data were normalized to β-actin control and calculated relative to untreated or vehicle control.

### Cell viability assays

Analysis of cell viability in PDOs or cell lines was performed as described previously [28, 30, 31]. Data were normalized to untreated control, set at 100% viability. For PDOs, data were calculated as the change in viability relative to control (set at 100%).

### Assessment of autophagic flux

The pBabe-puro retroviral expression vector was used to stably express mCherry-EGFP-LC3B. Autophagic flux was calculated as the ratio change in the median fluorescence intensity of mCherry:GFP as determined by flow cytometry [32].

### shRNA-mediated Knockdown of ATG5

Hec50 and SKOV3 cells were infected with lentivirus containing either nontargeting shRNA or shRNAs against ATG5 (TRCN0000151963 and TRCN0000151474 obtained from the RNAi Consortium).

### Animal studies

Animal studies were performed under animal protocol #0022285-003 approved by UI Institutional Animal Care and Use Committee. NOD.Cg-Prkdcscid Il2rgtm1Wjl/SzJ mice were subcutaneously injected with Hec50 cells and treated starting on day 18 after engraftment.

### Immunofluorescence imaging

Immunofluorescence staining was performed using 5 μm-thick sections of post-treatment tissue samples. Images were visualized by fluorescence microscopy at ×63 magnification.

## Results

### The majority of endometrial and ovarian cancer PDO models and cell lines are sensitive to dual treatment with a proteasome inhibitor and HDAC inhibitor

**Fig. 1:**
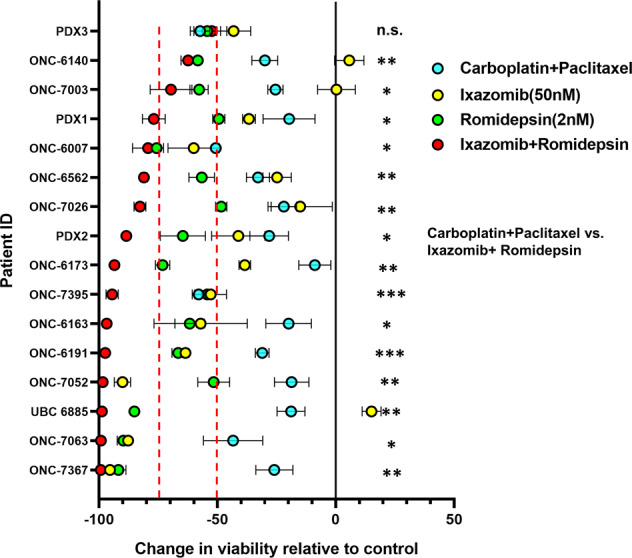
Most endometrial and ovarian cancer PDO models are highly sensitive to ixazomib and romidepsin as compared to standard chemotherapy. PDOs of fresh patient tumor specimens (indicated by Patient ID) or PDX models cultured in the organoid model system (PDX1-3) were treated with standard chemotherapy (1 µM carboplatin + 14 nM paclitaxel), ixazomib (50 nM), romidepsin (2 nM) or ixazomib + romidepsin for 72 h, followed by assessment of cell viability. Data were calculated as the change in viability relative to control, which was set at 100% (i.e., no cell death). Statistical significance was assessed by two-way ANOVA with Tukey’s post hoc test. Significant differences between carboplatin + paclitaxel vs. ixazomib + romidepsin are annotated for each PDO. n.s. not significant; *p < 0.05; **p < 0.01; ***p < 0.001. Patient information is provided in Supplementary Table S1. All statistical comparisons are provided in Supplementary Table S2.

To understand the effect of ixazomib and romidepsin on cell viability, drug response assays were performed on 17 PDO models of endometrial and ovarian cancer from patient tumor tissues as well as three endometrial cancer PDX models (Table S1). As compared to untreated controls, PDOs exhibited a notable decrease in viability when treated with ixazomib and romidepsin, with cell-killing ranging from 52.3 to 99.7% (Fig. 1). Similar results were obtained with other HDAC inhibitors and proteasome inhibitors (Fig. S1), indicating this effect is not specific to romidepsin and ixazomib. Models were also exposed to carboplatin plus paclitaxel, the most frequently used first-line chemotherapeutic regimen for gynecologic malignancies. Compared to untreated control, PDOs were differentially sensitive to standard chemotherapy, with decrease in viability from 8.8 to 57.9% (Fig. 1). Moreover, 16/17 PDOs were more sensitive to ixazomib and romidepsin as compared to standard chemotherapy (Table S2).

**Fig. 2:**
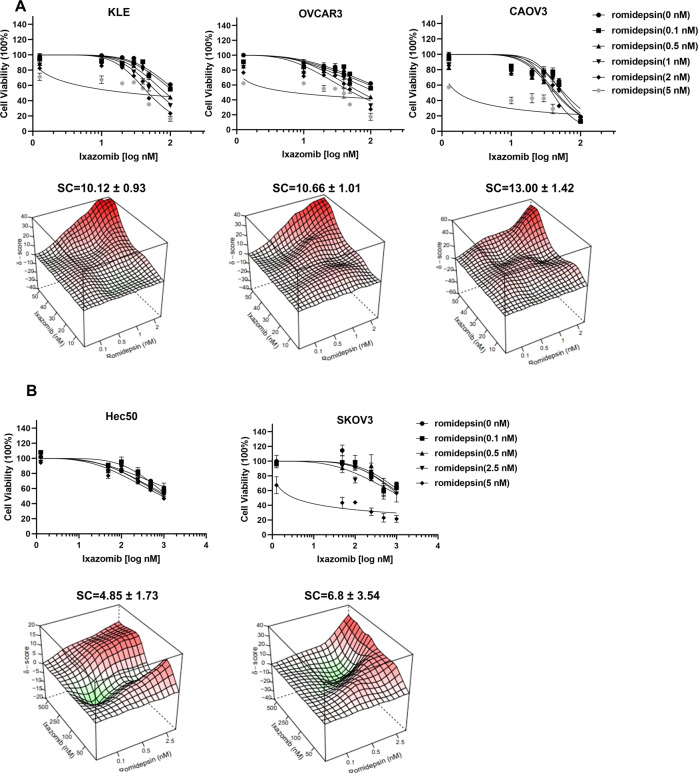
Gynecologic cancer cell lines display differential sensitivity to the combination of romidepsin and ixazomib. Data are separated into (**A**) sensitive cells (KLE, OVCAR3, and CAOV3) and (**B**) resistant cells (Hec50 and SKOV3) as determined by viability after treatment with romidepsin ± ixazomib for 72 h. The dose-dependent inhibition curves for drug combinations at varying concentrations are shown on the upper panels. Note that the dose-response curve for ixazomib alone (filled black circles) is denoted as “romidepsin (0 nM).” Lower panels are 3D plots used to determine synergy scores (SC). An SC of >10 indicates synergy.

Studies were extended to well-characterized advanced endometrial and ovarian cancer cell models. Synergy between ixazomib and romidepsin was assessed under varying drug concentrations by checkerboard assays followed by analysis using the highest single agent (HSA)-independent method. In KLE, OVCAR3, and CAOV3 cells, combining romidepsin with ixazomib produced a significant synergistic effect on cell killing (synergistic score >10; Fig. 2A) at nanomolar concentrations. However, in Hec50 and SKOV3 cells, combining romidepsin with ixazomib did not result in a synergistic response despite using higher drug concentrations (synergistic score <10; Fig. 2B).

### Ixazomib and romidepsin elevate autophagic flux in resistant cells

**Fig. 3:**
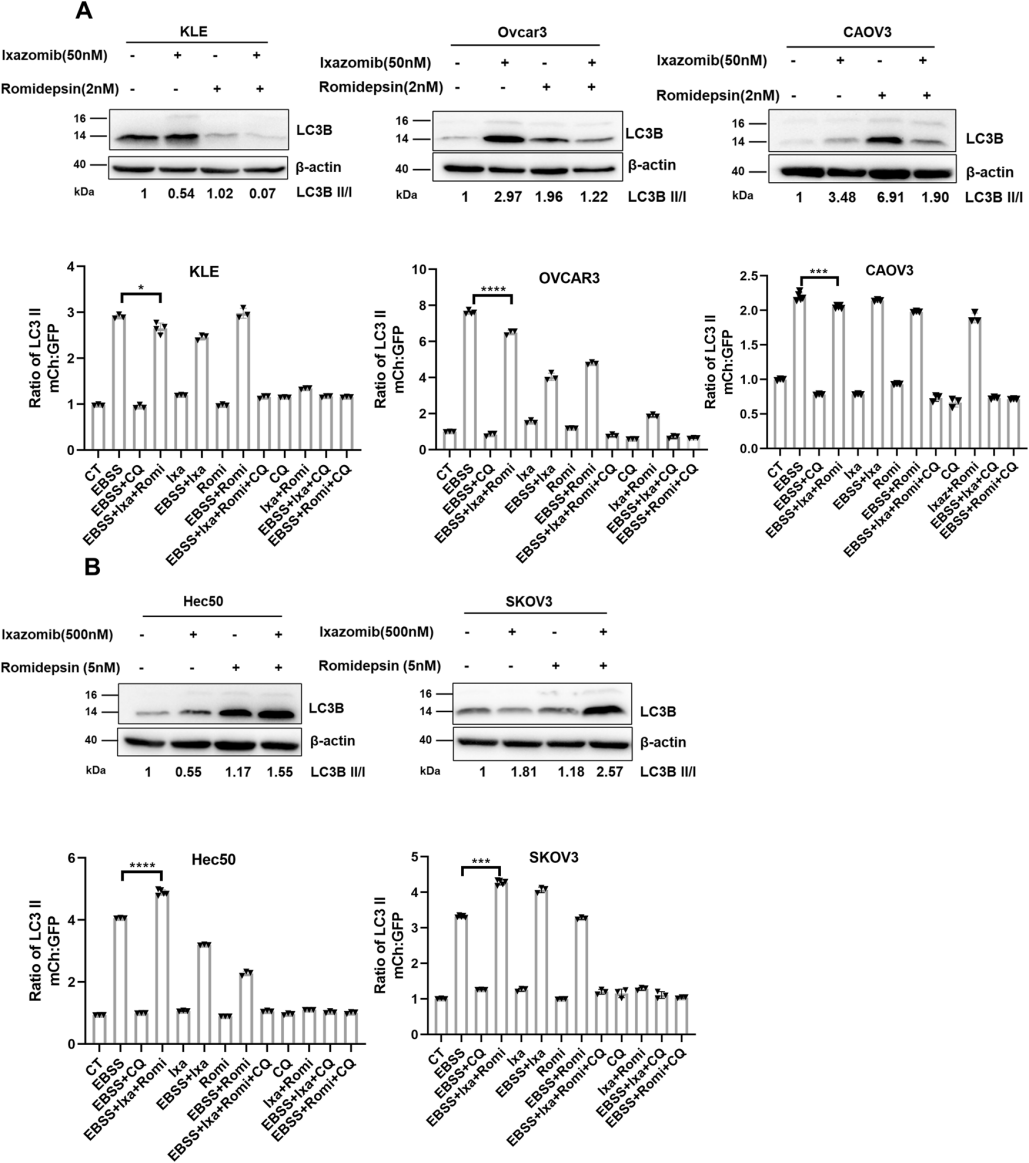
Ixazomib and romidepsin induce autophagy in resistant Hec50 and SKOV3 cells but not in sensitive cells. **A** Sensitive cells (KLE, OVCAR3, and CAOV3) or **B** resistant cells (Hec50 and SKOV3) were treated with indicated concentrations of ixazomib ± romidepsin for 72 h. BAF-A1 (200 nM) was added in the final 2 h prior to lysis. Upper panels: Western blots were performed to examine autophagic flux as indicated by the conversion of LC3B-I into LC3B-II. The ratio of LC3B-II/I, normalized to control (set at 1), is indicated for each treatment. Lower panels: Sensitive cells (KLE, OVCAR3, and CAOV3) or resistant cells (Hec50 and SKOV3) were transduced to stably express mCherry-EGFP-LC3B, and then exposed to either standard media or EBSS starvation media (to induce autophagy) in the absence or presence of ixazomib, romidepsin and chloroquine (CQ, 50 µM) for 18 h. Intensity of mCherry and EGF was determined by flow cytometry and autophagic flux calculated as the ratio of mCherry:GFP and normalized to DMSO control (set at 1). *p < 0.05; **p < 0.01; ***p < 0.001 *vs*. control by two-tailed unpaired Student’s *t* test.

Recent studies revealed that cancer cells utilize autophagy as a protective cellular survival and defense mechanism to maintain functional mitochondria, reduce DNA damage, and remain viable in response to stress. We hypothesized that resistance to ixazomib and romidepsin in Hec50 and SKOV3 cells is associated with activation of autophagic cell protective mechanisms. We thus assessed the conversion of LC3B-I to LC3B-II by Western blotting. This conversion represents a classic marker of autophagy. To detect autophagic flux, cells were treated with ixazomib and romidepsin 72 h followed by treatment with the autophagy inhibitor BAF-A1 for 2 h. In contrast to sensitive cells (KLE, OVCAR3 and CAOV3), LC3B-I to LC3B-II conversion increased in resistant cells (Hec50 and SKOV3, Fig. 3A, B, upper panel). These data confirm the induction of autophagy in resistant cells in response to treatment.

Using a pH-sensitive mCherry-GFP-LC3B tandem fluorescent reporter, we examined autophagic flux by flow cytometry. The GFP signal is quenched by the acidic environment of the autophagosome following fusion with the lysosome, whereas the mCherry signal is more stable [33]. Comparison of the ratio of the median fluorescence intensity of mCherry and GFP provides a quantitative measurement for autophagy [32]. Under nutrient-rich conditions, there was no measurable difference in autophagic flux with ixazomib and romidepsin treatment compared to control and single drug treatment (Fig. 3A, B, lower panels). In comparison, serum starvation with EBSS induced a significant increase in autophagy. The autophagy induced by starvation was decreased by ixazomib and romidepsin in the sensitive KLE, OVCAR3, and CAOV3 cells but increased in resistant Hec50 and SKOV3 cells (Fig. 3A, B, lower panel). We propose that increased autophagic flux in response to ixazomib and romidepsin treatment in Hec50 and SKOV3 cells indicates a signal of developing cellular resistance.

### Inhibition of autophagy sensitizes resistant cells to ixazomib and romidepsin in vitro

**Fig. 4:**
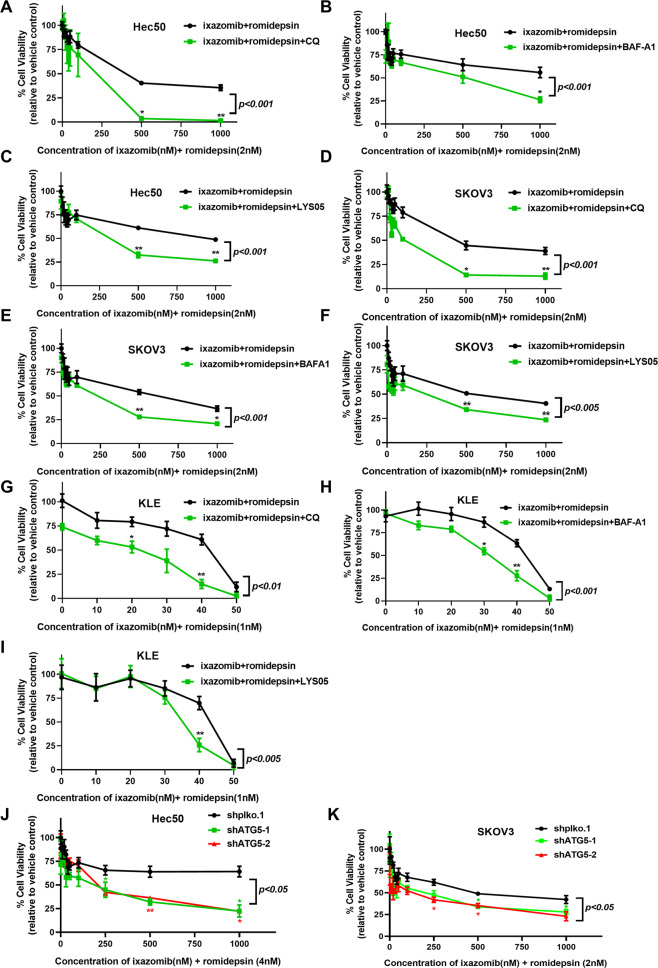
Inhibition of autophagy increases sensitivity to ixazomib and romidepsin in Hec50, SKOV3, and KLE cells. Hec50 cells were treated with ixazomib and romidepsin with or without autophagy inhibitors 50 µM CQ (**A**), 1.5 nM BAF-A1 (**B**) or 2 µM Lys05 (**C**) for 72 h. SKOV3 cells were treated with ixazomib and romidepsin with or without autophagy inhibitors 50 µM CQ (**D**), 1.5 nM BAF-A1 (**E**) or 2 µM Lys05 (**F**) for 72 h. KLE cells were treated with ixazomib and romidepsin with or without autophagy inhibitors 50 µM CQ (**G**), 1.5 nM BAF-A1 (**H**) or 2 µM Lys05 (**I**) for 72 h. Cell viability was determined using WST-1 assay. For Hec50 and SKOV3 cells, 2 nM romidepsin was set as control (100%); 1 nM romidepsin was set as control (100%) for KLE cells. Cell viability was assessed in Hec50 (**J**) and SKOV3 (**K**) cells with knockdown of ATG5 and treatment with ixazomib and romidepsin for 48 h. Cell viability was determined using WST-1 assay relative to untreated control. Statistical significance was assessed by two-way ANOVA with Sidak’s post hoc test. In addition, differences within a concentration were assessed by two-tailed unpaired Student’s *t* test. *p < 0.05; **p < 0.01 vs. ixazomib+romidepsin (**A**–**I**); *p < 0.05; **p < 0.01 vs. nontargeting shRNA (shplko.1).

To assess whether inhibition of autophagy increases the efficacy of ixazomib and romidepsin, we combined ixazomib and romidepsin with pharmacologic inhibitors of autophagy, chloroquine (CQ), BAF-A1 and LYS05. Autophagy inhibitors increased the sensitivity to ixazomib and romidepsin in previously resistant Hec50, SKOV3, and KLE cells (Fig. 4A–I), but not OVCAR3 and CAOV3 cells that initially demonstrated sensitivity to dual therapy (Fig. S2).

Because CQ, LYS05, and BAF-A1 may have effects on cell survival other than inhibition of autophagy, we also inhibited autophagy by knocking down ATG5, a critical protein required for autophagosome formation [34, 35]. ATG5 silencing is shown in Fig. S3. Hec50 and SKOV3 cells lacking ATG5 had enhanced sensitivity to ixazomib and romidepsin (Fig. 4J-K). These data support the induction of autophagy as a mediator of resistance to the combination of ixazomib and romidepsin.

**Fig. 5:**
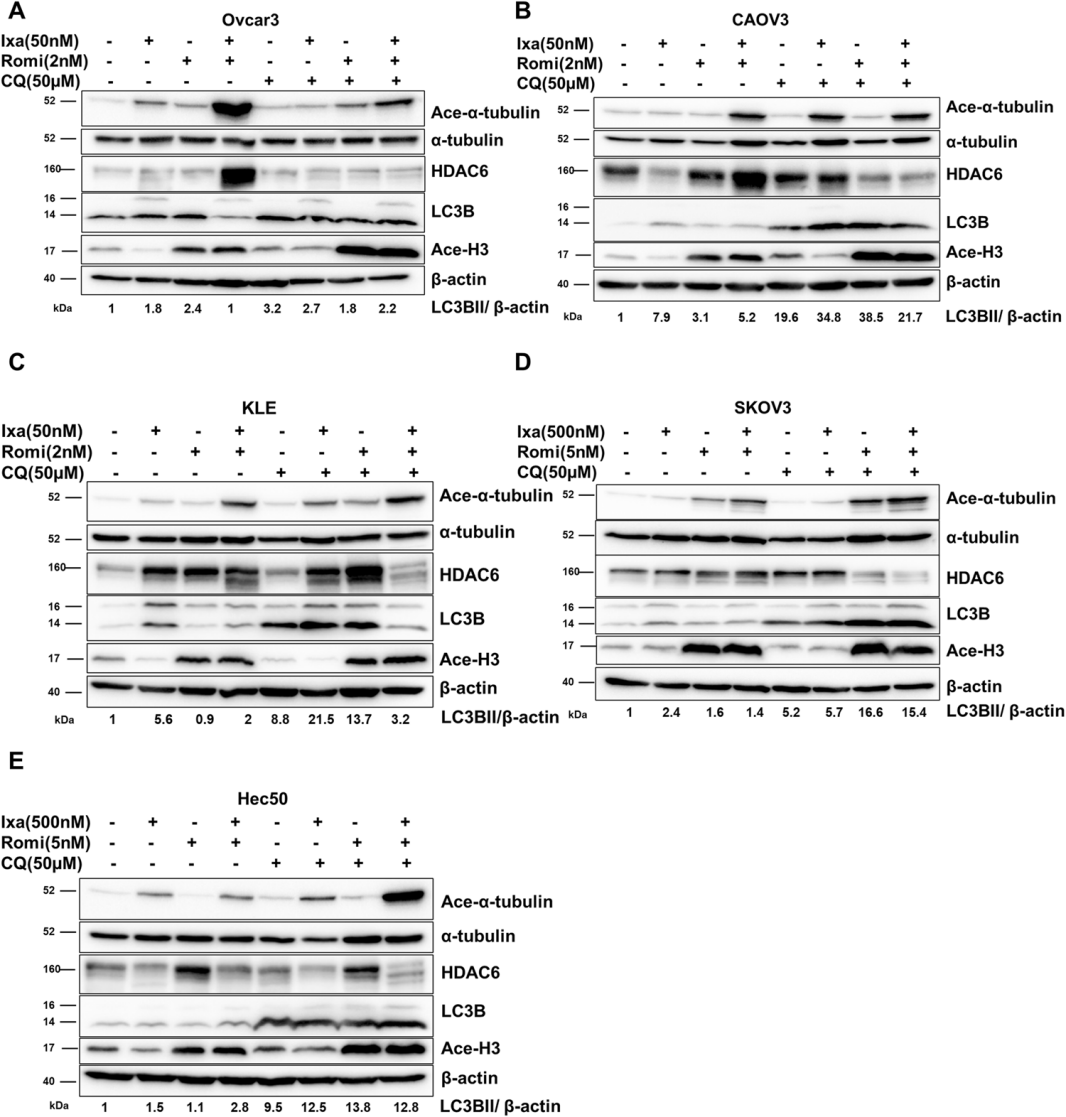
Autophagy inhibitors increase sensitivity to ixazomib and romidepsin by reducing HDAC6 activity in Hec50, SKOV3, and KLE cells. Ovcar3 (**A**), CAOV3 (**B**), KLE (**C**), SKOV3 (**D**) and Hec50 (**E**) cells were treated as indicated for 72 h, and then collected for Western blotting analysis of expression levels of acetylated (ace)-α-tubulin (a marker for HDAC6 inhibition), HDAC6, LC3B, and acetylated-histone H3 (Ace-H3, a marker for class I HDAC inhibition).

### Autophagy inhibition sensitizes resistant cells to ixazomib and romidepsin by inhibiting HDAC6 activity

Having established that the addition of autophagy inhibitors sensitizes resistant cells to ixazomib and romidepsin, we examined potential mechanism(s).

First, we confirmed induction of autophagy with ixazomib and romidepsin treatment in Hec50 and SKOV3 cells. CQ inhibits autophagy by blocking the fusion of the autophagosome with the lysosome, thereby inhibiting lysosomal-mediated protein degradation. LC3B-II on the surface of autophagosomes is normally degraded after fusion with the lysosome. Hence, after blocking the lysosome with CQ, increased LC3B-II expression following drug treatment indicates induction of autophagy, whereas decreased LC3B-II reflects a reduction [33].

Co-treatment of KLE, OVCAR3, and CAOV3 cells with CQ in combination with ixazomib and romidepsin results in decreased expression of LC3B-II compared to CQ alone (Fig. 5A–C), indicating reduced autophagy. In contrast, CQ, ixazomib and romidepsin treatment of Hec50 and SKOV3 cells increased LC3B-II compared to CQ alone, indicating the induction of autophagy (Fig. 5D, E). These results, taken together with those in Fig. 3, demonstrate that resistance of Hec50 and SKOV3 cells to ixazomib and romidepsin is mediated by increased autophagy.

We next considered the components of the autophagy pathway in order to more fully understand why certain cells may preferentially utilize autophagy as a survival mechanism. HDAC6 is an important regulator in the aggresome and autophagy pathway by chaperoning misfolded proteins to aggresomes for lysosomal degradation [36]. To understand the mechanism of resistance to ixazomib and romidepsin, we examined the acetylation of α-tubulin (ace-α-tubulin) after the inhibition of autophagy. A decrease in HDAC6 activity results in increased ace-α-tubulin. Treatment with ixazomib and romidepsin in combination with CQ did not increase ace-α-tubulin compared to ixazomib and romidepsin treatment in OVCAR3 and CAOV3 cells that do not mount an autophagy survival response and are therefore sensitive to proteasome plus HDAC inhibition (Fig. 5A, B). However, treatment of Hec50, SKOV3, and KLE cells with ixazomib and romidepsin in combination with CQ increased ace-α-tubulin, indicating HDAC6 inhibition (Fig. 5C–E). Since romidepsin is a specific Class I HDAC inhibitor, and HDAC6 belongs to the Class II HDAC family, we surmised that the inhibition of HDAC6 is not due to the direct effects of romidepsin on HDAC6. Supporting this notion, our data demonstrate a lack of change in ace-α-tubulin in cells treated with romidepsin alone. Increased acetylation of histone H3 confirms the romidepsin effect on Class I but not Class II HDACs [37] (Fig. 5).

We also detected cell-specific variations HDAC6 expression in response to treatment. HDAC6 was markedly increased in sensitive OVCAR3 and CAOV3 cells in response to combination treatment, potentially because these cells countered therapy by producing more HDAC6, but failed to survive regardless. Taken together, our findings indicate that CQ sensitizes Hec50, SKOV3, and KLE cells to ixazomib and romidepsin treatment by inhibiting the activity of HDAC6.

### Inhibition of autophagy with hydroxychloroquine sensitizes a resistant cell-derived xenograft model to ixazomib and romidepsin

**Fig. 6:**
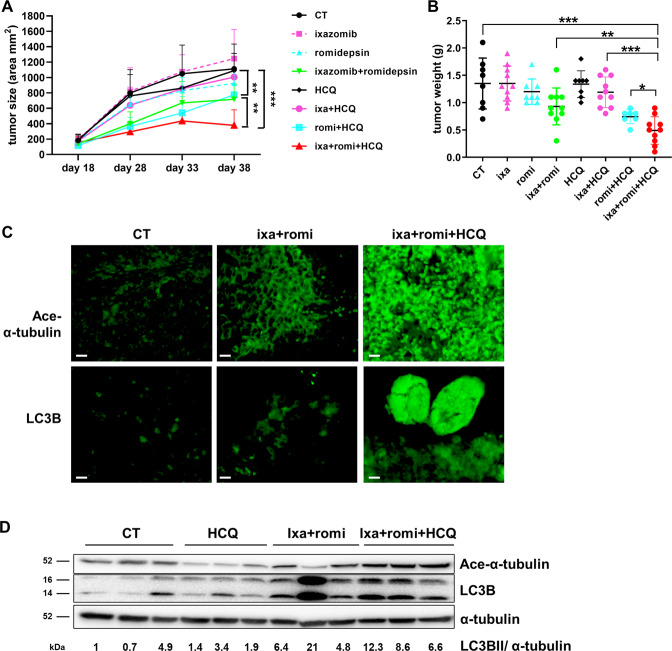
Hydroxychloroquine increases ixazomib and romidepsin sensitivity in Hec50-derived human endometrial cancer xenografts. **A** Growth curves for tumor volumes. CT control, ixa ixazomib, romi romidepsin, HCQ hydroxychloroquine. Statistical significance was assessed by two-way ANOVA with Sidak’s post hoc test. **p < 0.01; ***p < 0.001 as indicated. **B** Tumor weight was determined at the completion of treatment. Statistical significance was assessed by student’s *t*-test. *p < 0.05; **p < 0.01; ***p < 0.001 as indicated. **C** Expression of acetylated (ace)-α-tubulin and total LC3B was assessed in post-treatment tumor tissues by immunofluorescence imaging. Representative images are provided. CT control. Scale bar: 10 µm. **D** LC3B-I and -II and ace-α-tubulin were detected by Western blot analysis of post-treatment tumor lysates. Total α-tubulin levels serve as a loading control.

Given the enhanced cytotoxicity observed in cell cultures with ixazomib and romidepsin in combination with autophagy inhibitors, we asked whether autophagy inhibition augments the response to ixazomib and romidepsin in vivo using a xenograft model of the resistant cell line Hec50. Triple therapy of ixazomib, romidepsin, and hydroxychloroquine (HCQ) resulted in the strongest inhibition of tumor growth and the lowest tumor weight in vivo (Fig. 6A, B). Single drug treatments did not inhibit tumor growth in comparison with vehicle control, but a modest tumor growth inhibition was observed when romidepsin was combined with either ixazomib (*P* < 0.01) or HCQ (*P* < 0.05). The combination of romidepsin + ixazomib + HCQ also induced moderate body weight loss in mice (Fig. S4).

To assess molecular correlates of response, LC3B and ace-α-tubulin were examined in post-treatment tumor tissues by immunofluorescence (Fig. 6C) or Western blotting (Fig. 6D). A marked increase in LC3B-II and ace-α-tubulin was observed in tumors from animals treated with romidepsin + ixazomib in combination with HCQ, consistent with cell line data in Fig. 5. Increased expression of LC3B by immunofluorescence or the conversion of LC3B-I to LC3B-II by Western blotting in tumors treated with the dual or triple-drug combination reflects induction of autophagy. Similarly, we interpret the elevated acetylation of α-tubulin in tumors treated with romidepsin + ixazomib + HCQ as evidence for greater HDAC6 inhibition in response to the triple-drug regimen as compared with romidepsin + ixazomib.

## Discussion

In this study, we report for the first time that the combination of ixazomib and romidepsin induces significant cell death in many gynecologic cancer PDOs and cell lines. This was somewhat surprising, as most HDAC and proteasome inhibitors have not been deemed active as single agents in clinical studies of gynecologic cancers [9–11] or in combination with chemotherapy [11, 15]. Supporting this, we saw wide variability in the PDO models to individual HDAC and proteasome inhibitory agents, but the combination was highly effective in majority of models. The PDO culture method provides a novel and powerful platform for studying patient tumors in vitro. Accumulating evidence has emerged that PDOs can predict clinical outcomes in many types of cancers. A previous report from our laboratory substantiated that PDOs have the potential to predict patient responses to chemotherapy [28]. In the current study, the most impressive effects were observed with the combination of HDAC and proteasome inhibitors, especially when compared to standard chemotherapy. Given the increasing recognition that PDOs are reliable surrogates of patient response, our data provide a rationale to pursue the combination of ixazomib and romidepsin clinically despite the perceived failure of single-agent clinical trials.

One of the most important and unexpected findings from this study is that some gynecologic cancer models induce autophagy as a protective mechanism to sustain viability when exposed to proteasome and HDAC inhibitors. Inhibition of autophagy significantly enhances the synergistic effect of the combination therapy in resistant cell lines as well as a xenograft model in vivo. These preclinical findings for the first time identify autophagy as a biomarker of resistance.

As an evolutionarily conserved degradation process, autophagy eliminates unfolded/misfolded/aggregated proteins and damaged organelles in response to stress or starvation [38]. While the original dogma was that autophagy was a mechanism of cell death, accumulating evidence suggests that autophagy also facilitates survival in response to cellular stresses such as hypoxia, DNA damage, and chemotherapy [39, 40]. Our data add to this body of literature by demonstrating that treatment with an HDAC and proteasome inhibitor also induces autophagy-mediated cell survival. Although preclinical data have supported the use of an HDAC inhibitor in combination with a proteasome inhibitor in some cancers, the role of autophagy in response to this combination has not been fully explored. Moreover, the results of the limited studies that have addressed this question are conflicting. Autophagy is widely thought to contribute to proteasome inhibitor resistance by providing a compensatory mechanism for dysfunctional protein clearance [41]. For example, the combination of bortezomib and romidepsin in gastric carcinoma induces autophagy, which the authors concluded was a mechanism of cell death [42]. In head and neck squamous cell carcinoma, trichostatin A reduces bortezomib-induced autophagy, and the combination promotes cell death [43]. Treatment with an HDAC inhibitor activates varying molecular mechanisms that can lead to either the activation or suppression of autophagy [44, 45] To our knowledge, our study is the first to report the role of autophagy in regulating sensitivity to the combination of HDAC and proteasome inhibitors in gynecologic cancers.

In sensitive cell lines, treatment with either an HDAC or proteasome inhibitor alone increased conversion of LC3I to LC3II, whereas the dual treatment did not produce such an effect. We interpret this increase in LC3 conversion as a compensatory mechanism of cellular preservation. In the setting of the dual drug treatment, this is not sufficient to overcome the cytotoxic effects of drugs. Consistent with this interpretation, we observe enhanced cell killing with the double-drug combination vs. single drug for all sensitive cells.

**Fig. 7:**
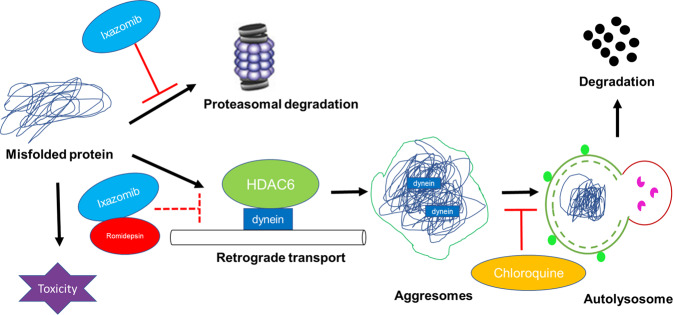
Schematic depicting the proposed effects of ixazomib, romidepsin, and chloroquine on the proteasome and autophagy signaling pathways.

From the perspective of mechanism, we propose that HDAC6 is a critical mediator of resistance through induction of autophagy (Fig. 7). HDAC6 along with the dynein complex recruits and transports misfolded proteins to aggresomes/autophagosomes via the microtubule network for subsequent degradation by lysosomes (Fig. 7). Inhibiting HDAC6 blocks the autophagy pathway in many cancer types [43, 46, 47]. Thus, HDAC6 has been implicated as a potential target to overcome proteasome inhibitor resistance. Although many HDAC6-selective small-molecule inhibitors have been discovered, not all of them modulate autophagy. No specific inhibitor of HDAC6 has yet been approved for clinical use. Remarkably, in this study, the combination of ixazomib and romidepsin, a Class I HDAC inhibitor that does not directly inhibit HDAC6 (a Class II HDAC [37, 48]), significantly inhibited HDAC6 activity. In sensitive cells, adding an autophagy inhibitor had no additional effect. However, in resistant cells, adding an autophagy inhibitor to the combination of ixazomib and romidepsin further reduced the activity of HDAC6 (as evidenced by increased ace-α-tubulin), leading to cell death. These findings suggest that HDAC6 inhibition can be achieved by treatment with the combination of a proteasome inhibitor and a class I HDAC inhibitor such as romidepsin.

Limitations of this study include the use of autophagy inhibitors that have pleotropic effects. However, the concept that adaptive resistance is mediated by autophagy was confirmed in our studies by demonstrating similar effects using multiple agents and methods, including ATG5 silencing. One potential explanation for the differential sensitivity of the various cell lines is reliance on autophagy for survival in response to cellular stress. For example, the sensitive line OVCAR3 is dependent upon high glucose for survival, whereas the resistant SKOV3 is a low glucose-dependent cell line [49]. Hence, it is possible that SKOV3 cells have an inherent reliance on the autophagy pathway to preserve cellular nutrients. The glucose dependence of the other resistant line, Hec50, has not been investigated.

Another limitation is the lack of a pretreatment biomarker that distinguishes between cells that induce autophagy in response to the combinatorial regimen vs. those that do not. We believe that autophagy is a mechanism that activated in response to drug; thus, that baseline expression of autophagy factors such as LC3B may not be an adequate predictor of sensitivity to therapy. This is the reason we performed the assessment of autophagy biomarkers in the control vs. treated tumor specimens from the xenograft models. This work highlights the need to derive and approve biomarkers for autophagy that can serve as reliable translational endpoints for future trials.

In conclusion, these preclinical studies defined the efficacy of combining a proteasome and HDAC inhibitor in gynecologic cancer cells. Our results suggest that the combination of ixazomib and romidepsin is a possible novel strategy to improve the outcomes of patients with gynecologic cancer, and adding an autophagy inhibitor to this combination in the future may be an option to overcome resistance.

## Supplementary information

Supplemental Material and Methods

Checklist

## Data Availability

All data are included in this manuscript or the supplemental materials.
