# Supplementary material for: Blocking autophagy overcomes resistance to dual histone deacetylase and proteasome inhibition in gynecologic cancer
Source: Cell Death Dis. 2022 Jan 17;13(1):59. doi: 10.1038/s41419-022-04508-2 (PMC8763941; doi:10.1038/s41419-022-04508-2)
Supplement: Supplementary file 1 — Supplemental Material and Methods [file 41419_2022_4508_MOESM1_ESM.pdf]

Supplemental Material for Bi et al, "Blocking autophagy overcomes resistance to dual histone deacetylase and proteasome inhibition in gynecologic cancer"

Contents:

Supplemental Methods

Figure S1. Endometrial and ovarian cancer PDO models are highly sensitive to proteasome inhibitor bortezomib and HDAC inhibitor belinostat.

Figure S2. Inhibition of autophagy has no effect on sensitivity to ixazomib and romidepsin treatment in OVCAR3 and CAOV3 cells.

Figure S3. Knockdown of ATG5 in Hec50 and SKOV3 cells

Figure S4. Body weight of xenograft tumors

Table S1. Patient characteristics corresponding to PDOs used for drug screening studies.

Table S2. Statistical comparisons of single agents vs. control or dual treatments for Figure 1 and Figure S1.

Supplemental Methods

Chemicals

For *in vitro* experiments, ixazomib, romidepsin, chloroquine, bafilomycin A1 and lys05 were purchased from Selleck Chemicals, LLC (Houston, TX, USA) and suspended in DMSO. For *in vivo* studies, ixazomib citrate was dissolved in 5% 2-hydroxypropyl-β-cyclodextrin (Sigma Chemical Company, St. Louis, MO, USA), romidepsin was dissolved in 2% DMSO, 30% 400PEG, 5% Tween 80 in ddH20, hydroxychloroquine was dissolved in 0.9% sodium chloride.

Patient-derived Organoid Models

All studies using human tissues have been approved by the University of Iowa Institutional Review Board (IRB), protocol #201809807. Patient tumor specimens were obtained, processed, and cultured to create PDO cultures per our previously published protocol [1]. Briefly, tumor tissues were minced and dissociated to single cells. Equal numbers of cells were embedded in Matrigel, plated on pre-warmed 24-well cell culture plates and incubated at 37°C for 15 min to allow Matrigel to solidify. Organoid culture media [1,2] was then added to each well. All experiments were performed within two weeks of organoid generation.

Cell Culture

Hec50 cells were kindly provided by Dr. Erlio Gurpide (New York University, New York, NY, USA) [3] and grown in high-glucose Dulbecco's Modified Eagle Medium (DMEM; Gibco Corporation, Gaithersburg, MD, USA) supplemented with 10% fetal bovine serum (FBS). HEK293T cells were purchased from ATCC and grown in DMEM medium supplemented with 10% FBS. KLE, OVCAR3, CAOV3 and SKOV3 cells were purchased from ATCC and grown in RPMI-1640 medium supplemented with 20% FBS. To ensure rigor and reproducibility, the

identity of all cell lines was confirmed using the CODIS genotyping test (Cat. No. CL1003, Bio-Synthesis, Lewisville, TX, USA) [3].

Western Blotting

Cells or tumor tissues were collected and lysed with RIPA buffer (50 mM Tris-HCl, pH 7.4, 150 mM NaCl, 2 mM EDTA, 1% NP-40, 0.1% sodium dodecyl sulfate (SDS), protease inhibitors) as described previously [4]. The following antibodies from Cell Signaling (Danvers, MA, USA) were used at the indicated dilutions: anti-acetylated (ace)-α-tubulin (1:1000, #5335), anti-α-tubulin (1:1000, #2144), anti-HDAC6 (1:1000, #7558), anti-LC3B (1:1000, #3868), anti-acetylated (ace)-histone H3 (1:1000, #13998) and anti-ATG5 (1:1000, #12994). Anti-β-actin (1:10,000, sc-47778) was from Santa Cruz Biotechnology (Dallas, TX, USA). Signal bands were detected using the Bio-Rad ChemiDoc system and densitometry was analyzed with Bio-Rad Image Lab Software (Bio-Rad Laboratories, Redmond, WA, USA). Data were normalized to β-actin control and calculated relative to untreated or vehicle control (set at 1).

Cell Viability Assays

Viability of PDOs following drug treatment was performed as previously described using CellTiter-Glo 3D reagent (Promega, Madison, WI, USA) [1, 2]. Cell viability for cell lines was determined by WST-1 assay as previously described [5]. All experiments include three technical replicates. Data were normalized to untreated control, set at 100% viability. For organoid experiments, the data were calculated as the change in viability relative to control (set at 100%).

Retroviral Expression of mCherry-EGFP-LC3B

The pBabe-puro retroviral expression vector was used to ectopically express mCherry-EGFP-LC3B in endometrial and ovarian cancer cell lines. To generate retroviral particles, 1×10^5 low

passage number HEK293T cells were plated in 6-well plates and incubated overnight. HEK293T cells were then co-transfected with 2 µg pCL-10A1(NBP2-29542, NOVUS) and 2 µg pBABE-puro mCherry-EGFP-LC3B (22418, Addgene, Cambridge, MA, USA) using Lipofectamine 2000 (Invitrogen, Eugene, OR, USA) according to the manufacturer's protocol. Retroviral particles were collected from the media 48 h post-transfection, supplemented with 10 µg/ml polybrene and stored at -80°C. Cancer cells were transduced with retrovirus-containing media for 24 h then selected for puromycin resistance. Studies were performed in pooled populations of puromycin-resistant cells.

shRNA-mediated Knockdown of ATG5

Low passage number HEK293T cells were transfected with the PLKO.1 vector containing nontargeting shRNA or shRNAs against ATG5 (TRCN0000151963 and TRCN0000151474 obtained from the RNAi Consortium), along with PAX2 and VSVG, using Lipofectamine 2000 according to the manufacturer's protocol. Lentiviral particles were collected 48 h post-transfection, supplemented with 10µg/ml polybrene and stored at -80°C. Hec50 and SKOV3 cells ($1x10^5$) were seeded in 6-well plates, infected with lentivirus for 24 h then selected for puromycin resistance (10 µg/ml) for one week. The level of ATG5 knockdown in cells infected with nontargeting or ATG5-specific shRNAs was determined by Western blotting.

Flow Cytometry Analysis

Cells constitutively expressing mCherry-GFP-LC3B were plated at 2×10^5 cells/well in 6-cm dishes and incubated for 24 h at 37°C in 5% CO_2. Cells were then exposed to either the standard media or Earl's Balanced Salt Solution (EBSS) starvation media (#2888, Sigma, St. Louis, MO, USA) in the absence or the presence of drug as indicated. Flow cytometry was performed using the BD FACS Canto II equipped with a 561 nm laser for mCherry excitation and 488 nm laser

for GFP excitation. Autophagic flux was determined as the ratio change in the median fluorescence intensity of mCherry: GFP [6].

Animal Studies

All animal studies were performed under animal protocol #0022285-003 approved by the University of Iowa Institutional Animal Care and Use Committee (Iowa City, IA, USA). NOD.Cg-Prkdcscid Il2rgtm1Wjl/SzJ (NSG) immunodeficient mice (Jackson Laboratories, Bar Harbor, ME, USA) at 7-8 weeks of age were injected with Hec50 cells ($5x10^6$/100 µl PBS) into the right flank subcutaneously. NSG mice were randomized into 8 groups for treatment as follows: 1) control (5% 2-hydroxypropyl-β-cyclodextrin by gavage, 2% DMSO, 30% 400PEG, 5% Tween 80 in ddH_20 by IP); 2) hydroxychloroquine (HCQ) alone; 3) Ixazomib alone; 4) romidepsin alone; 5) a combination of ixazomib and romidepsin; 6) a combination of ixazomib and HCQ; 7) a combination of romidepsin and HCQ; 8) a combination of ixazomib, romidepsin and HCQ. Treatment was initiated on day 18 after engraftment of cells, at the time when tumors were detectable by palpation, and continued for three weeks. Ixazomib citrate (5 mg/kg) was administered by oral gavage twice a week. Romidepsin (1 mg/kg) was administered by intraperitoneal (IP) injection three times a week. Hydroxychloroquine (60 mg/kg) was administered 6 days per week via IP injection. Tumors were measured using calipers, and volumes were calculated using the formula length × $width^2$ / 2. Tumors were excised at the end of the treatment period for analysis by Western blotting or immunofluorescence imaging.

Immunofluorescence Imaging

Immunofluorescence staining was performed using 5 µm-thick sections of 4% paraformaldehyde-fixed paraffin-embedded post-treatment tissue samples. Next, sections were

permeabilized with 1% Triton X-100, labeled with an ace-α-tubulin (1:500, #5335, Cell Signaling) or a LC3B primary antibody (1:1000, #3868, Cell Signaling), and incubated with Alexa Fluor-488-conjugated secondary antibody (1:200, #A-11001, Invitrogen). Images were visualized by fluorescence microscopy and acquired with an Olympus BX51 camera at 63× magnification.

Statistical Analysis

Data were analyzed using GraphPad Prism software (GraphPad Software Version 0.0.0 (121), San Diego, CA, USA). Statistical significance of differences was determined using two-way analysis of variance (ANOVA) with Tukey's post-hoc test (for comparisons of means of multiple groups**; Figure 1**) or Sidak's multiple comparisons test (for comparison of significance between two treatment groups with multiple independent treatment concentrations, **Figures 2, 4, S1**). All values are expressed as mean ± standard deviation (SD) of at least three independent experiments unless otherwise indicated.

References for Supplemental Methods

1 Bi, J. *et al.* Successful Patient-Derived Organoid Culture of Gynecologic Cancers for Disease Modeling and Drug Sensitivity Testing. *Cancers (Basel)* **13**, doi:10.3390/cancers13122901 (2021).

2 Bi, J. *et al.* Characterization of a TP53 somatic variant of unknown function from an ovarian cancer patient using organoid culture and computational modeling. *Clin Obstet Gynecol* **63**, 109-119, doi:10.1097/GRF.0000000000000516 (2020).

3 Devor, E. J., Gonzalez-Bosquet, J., Thiel, K. W. & Leslie, K. K. Genomic characterization of five commonly used endometrial cancer cell lines. *Int J Oncol* **57**, 1348-1357, doi:10.3892/ijo.2020.5139 (2020).

4 Bi, J. *et al.* MTDH/AEG-1 downregulation using pristimerin-loaded nanoparticles inhibits Fanconi anemia proteins and increases sensitivity to platinum-based chemotherapy. *Gynecologic oncology* **155**, 349-358, doi:10.1016/j.ygyno.2019.08.014 (2019).

5 Bi, J. *et al.* Metadherin enhances vulnerability of cancer cells to ferroptosis. *Cell Death Dis* **10**, 682, doi:10.1038/s41419-019-1897-2 (2019).

6 Zahedi, S. *et al.* Effect of early-stage autophagy inhibition in BRAF(V600E) autophagy-dependent brain tumor cells. *Cell Death Dis* **10**, 679, doi:10.1038/s41419-019-1880-y (2019).

Figure S1

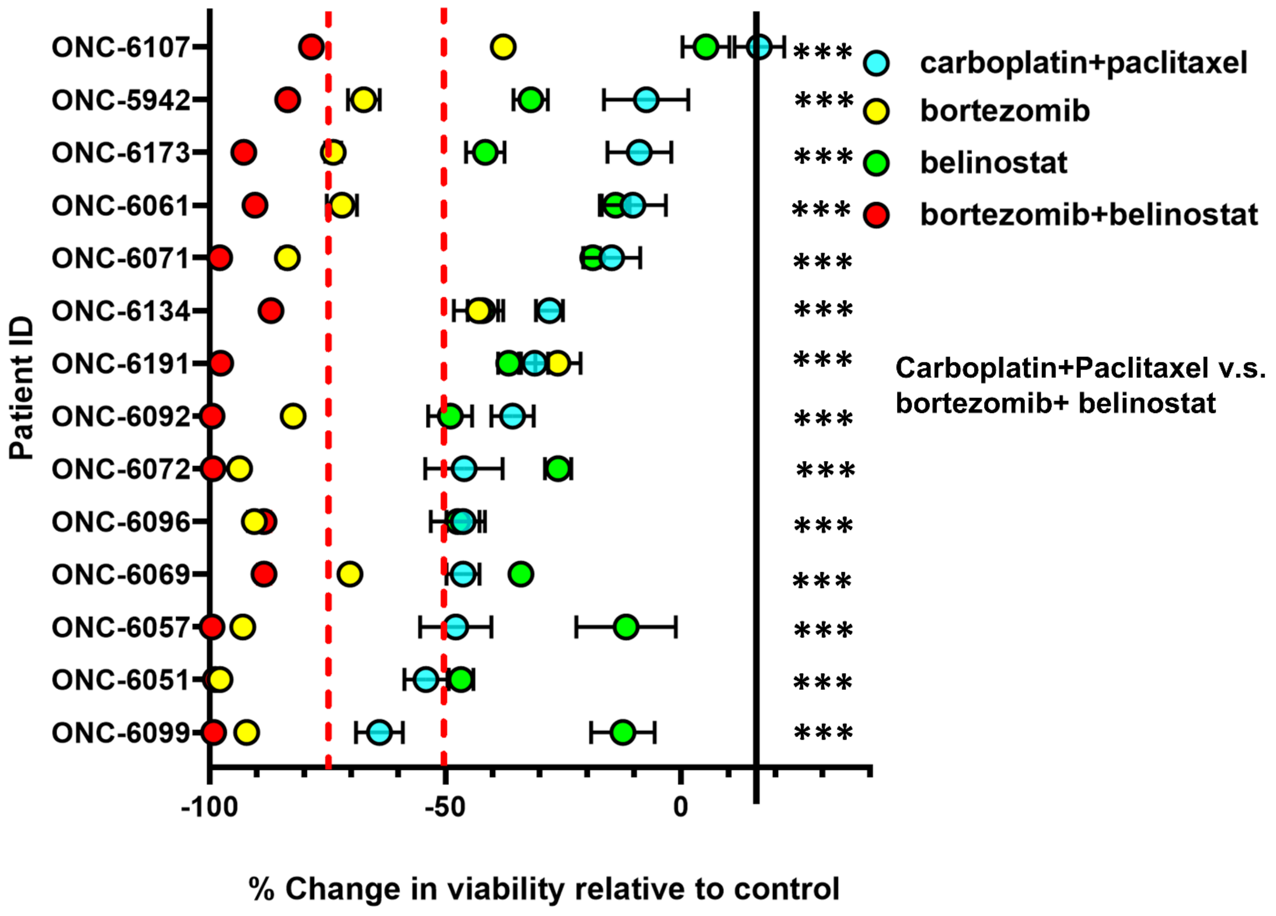

Figure S1. Endometrial and ovarian cancer PDO models are highly sensitive to proteasome inhibitor bortezomib and HDAC inhibitor belinostat. PDOs of fresh patient tumor specimens (indicated by Patient ID) were treated with standard chemotherapy (1 µM carboplatin + 14 nM paclitaxel), belinostat (1 µM), bortezomib (2 nM) or belinostat + bortezomib for 72 h, followed by assessment of cell viability. Data were calculated as the change in viability relative to control, which was set at 100% (i.e., no cell death). Statistical significance was assessed by two-way ANOVA with Tukey's post-hoc test. Significant differences between carboplatin + paclitaxel vs. bortezomib + belinostat are annotated for each PDO. n.s.: not significant; * $p<0.05$; ** $p<0.01$; *** $p<0.001$. All statistical comparisons are provided in Supplemental Table S1. Patient information is provided in Supplemental Table S2.

Figure S2

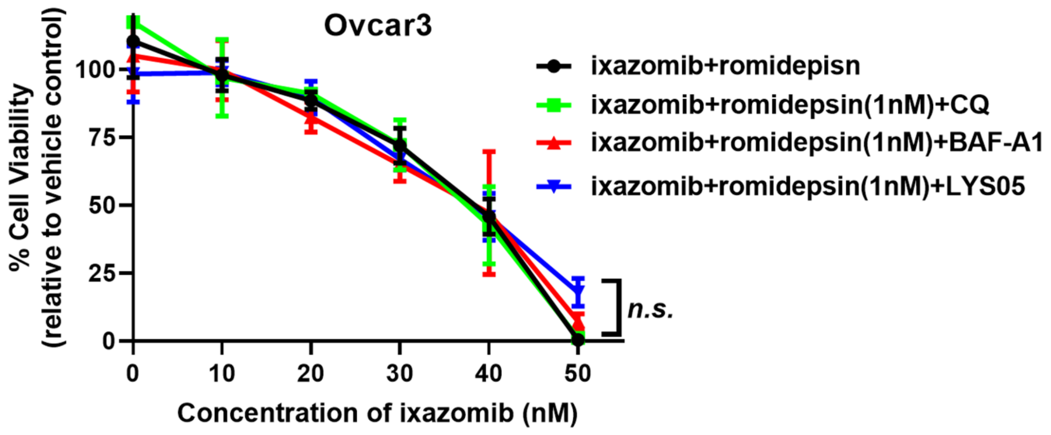

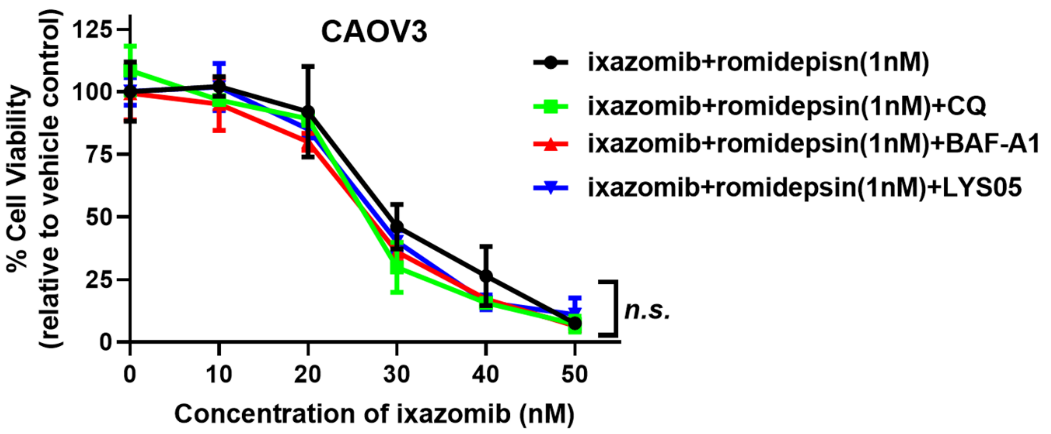

Figure S2. Inhibition of autophagy has no effect on sensitivity to ixazomib and romidepsin treatment in OVCAR3 and CAOV3 cells. Cells were seeded onto 96 well plates then treated with ixazomib and romidepsin with or without autophagy inhibitors (50µM CQ, 1.5nM BAF-A1 and 2µM Lys05) for 72 h; cell viability was determined using WST-1 assay wherein 1 nM romidepsin was set as control (100%). Statistical significance was assessed by two-way ANOVA with Sidak's post-hoc test. n.s.: not significant.

Figure S3

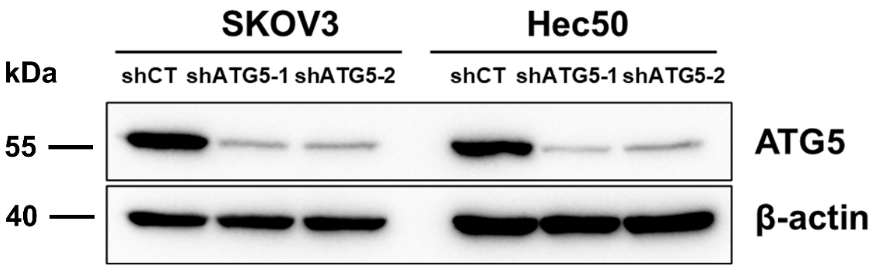

Figure S3. Knockdown of ATG5 in Hec50 and SKOV3 cells was confirmed by Western blotting.

Figure S4

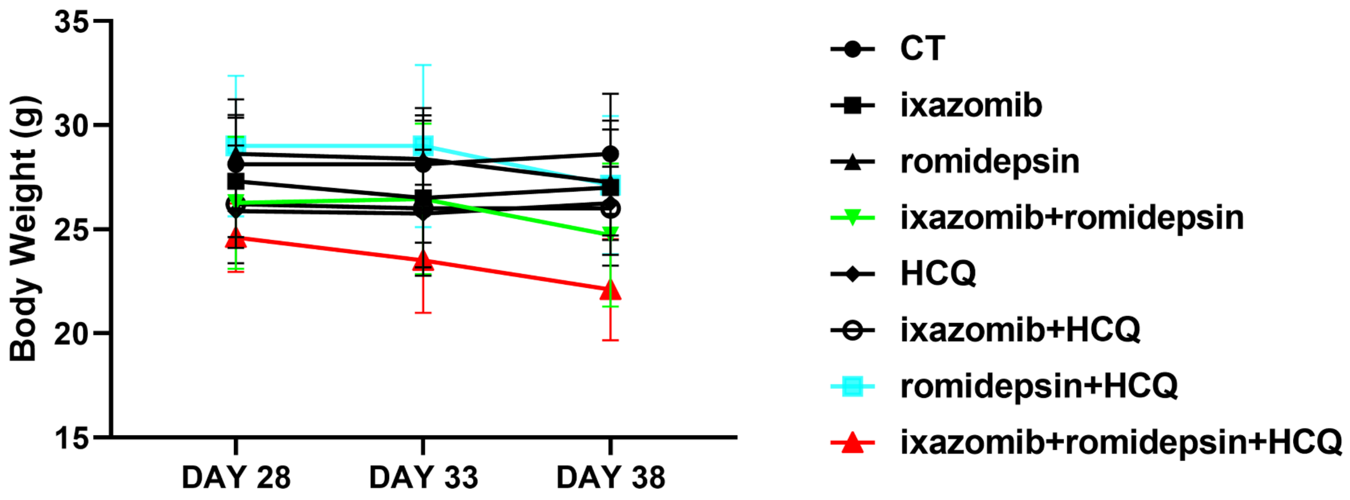

Figure S4. Body weight by treatment with vehicle control, ixazomib, romidepsin, hydroxychloroquine or their combination.

Table S1. Patient characteristics corresponding to PDOs used for drug screening studies. Note that some of these models have been studied previously by our group [28].

	Gyn-onc ID	Cancer Type	Stage	Adjuvant Therapy	Disease Status
ovarian	UBC 6885	Endometrial adenocarcinoma	IIIA	Chemotherapy	Need to follow up
ovarian	ONC-7052	High-grade serous	IIIC	HRT and chemotherapy	NED
ovarian	ONC-7063	Clear cell	IC1	Chemotherapy	NED
ovarian	ONC-6163	High-grade serous	Recurrent	Chemotherapy, PARP inhibitor, Lynparza	NED
ovarian	ONC-7395	Mucinous adenocarcinoma	IA	Chemotherapy	Need to follow up
ovarian	ONC-6562	High-grade serous	IVA	Chemotherapy and niraparib	Need to follow up
ovarian	ONC-6007	High-grade serous	IVB	Chemotherapy (Doxil/Carbo) and Avastin	NED
ovarian	ONC-6140	High-grade serous		Not indicated	Deceased
ovarian	ONC-6730	Endometrial adenocarcinoma	IA	Not indicated	NED
Ovarian	ONC-6072	High grade serous	IIIB	Chemotherapy, abraxane, avastin, and radiation therapy	Deceased
Ovarian	ONC-6069	High grade serous	IIIB	Chemotherapy, pelvic radiation, Lynparza, and Gemzar	Deceased
Ovarian	ONC-6061	Low grade serous carcinoma	IIIC	Chemotherapy and letrozole	NED
Ovarian	ONC-6092	Clear cell	IC3	Chemotherapy	NED
Ovarian	ONC-5942	High grade serous	IV	Chemotherapy, avastin/letrozole	Need to follow up
Ovarian	ONC-6134	High grade serous	IIIA	Chemotherapy	Need to follow up
Ovarian	ONC-6107	Mucinous borderline	IA	Not indicated	NED
Uterine	ONC-7026	Endometrioid	IA	Not indicated	NED
Uterine	ONC-7367	Clear cell	IB	Whole pelvic radiation	Need to follow up
Uterine	ONC-6191	Endometrioid	IB	Not indicated	NED
Uterine	PDX1	Serous adenocarcinoma			
Uterine	PDX2	High-grade endometrioid adenocarcinoma			
Uterine	PDX3	Low-grade endometrioid adenocarcinoma			
Uterine	ONC-6071	Endometrial adenocarcinoma	IA	Not indicated	Need to follow up
Uterine	ONC-6057	Serous	IVB	Chemotherapy and Avastin	Deceased
Uterine	ONC-6099	Serous	IVB	Chemotherapy, avastin, and radiation therapy	Deceased
Uterine	ONC-6096	Endometrioid	IA	Not indicated	N.E.D.
Uterine	ONC-6051	Endometrioid	IA	Not indicated	NED

Table S2 Statistical comparisons of single agents vs. control or dual treatments for Figure1 and Figure S1

Tukey's multiple comparisons test	Mean Diff.	95.00% CI of diff.	Significant?	Summary	Adjusted P Value
UBC 6885					
carbo+taxol vs. ixazomib(50nM)	-34.03	-52.03 to -16.03	Yes	**	0.0066
carbo+taxol vs. romidepsin(2nM)	66.14	42.89 to 89.38	Yes	**	0.0064
carbo+taxol vs. ixazomib+romidepsin	79.88	56.23 to 103.5	Yes	**	0.0044
ixazomib(50nM) vs. romidepsin(2nM)	100.2	84.75 to 115.6	Yes	****	<0.0001
ixazomib(50nM) vs. ixazomib+romidepsin	113.9	97.92 to 129.9	Yes	****	<0.0001
romidepsin(2nM) vs. ixazomib+romidepsin	13.75	11.38 to 16.11	Yes	***	0.0002
ONC-7026					
carbo+taxol vs. ixazomib(50nM)	-6.869	-52.03 to 38.30	No	ns	0.8452
carbo+taxol vs. romidepsin(2nM)	26.47	9.124 to 43.81	Yes	*	0.0168
carbo+taxol vs. ixazomib+romidepsin	60.81	43.62 to 77.99	Yes	**	0.0017
ixazomib(50nM) vs. romidepsin(2nM)	33.33	-18.29 to 84.95	No	ns	0.1177
ixazomib(50nM) vs. ixazomib+romidepsin	67.67	16.26 to 119.1	Yes	*	0.0285
romidepsin(2nM) vs. ixazomib+romidepsin	34.34	26.20 to 42.48	Yes	***	0.0002
ONC-7052					
carbo+taxol vs. ixazomib(50nM)	71.47	48.11 to 94.82	Yes	**	0.0025
carbo+taxol vs. romidepsin(2nM)	32.93	9.527 to 56.32	Yes	*	0.0157
carbo+taxol vs. ixazomib+romidepsin	79.8	50.67 to 108.9	Yes	**	0.0071
ixazomib(50nM) vs. romidepsin(2nM)	-38.54	-59.70 to -17.38	Yes	**	0.0095
ixazomib(50nM) vs. ixazomib+romidepsin	8.336	-5.246 to 21.92	No	ns	0.1249
romidepsin(2nM) vs. ixazomib+romidepsin	46.88	20.04 to 73.71	Yes	*	0.0169
ONC-7063					
carbo+taxol vs. ixazomib(50nM)	44.3	-5.308 to 93.91	No	ns	0.0622
carbo+taxol vs. romidepsin(2nM)	46.36	-0.6392 to 93.36	No	ns	0.0514
carbo+taxol vs. ixazomib+romidepsin	55.85	5.788 to 105.9	Yes	*	0.0405
ixazomib(50nM) vs. romidepsin(2nM)	2.058	-6.454 to 10.57	No	ns	0.6227
ixazomib(50nM) vs. ixazomib+romidepsin	11.55	8.231 to 14.86	Yes	**	0.0026
romidepsin(2nM) vs. ixazomib+romidepsin	9.489	-0.3581 to 19.34	No	ns	0.0538
ONC-6173					
carbo+taxol vs. ixazomib(50nM)	29.56	6.563 to 52.56	Yes	*	0.0277
carbo+taxol vs. romidepsin(2nM)	64.28	42.34 to 86.22	Yes	**	0.003
carbo+taxol vs. ixazomib+romidepsin	84.65	57.98 to 111.3	Yes	**	0.005
ixazomib(50nM) vs. romidepsin(2nM)	34.72	25.40 to 44.05	Yes	***	0.0005

ixazomib(50nM) vs. ixazomib+romidepsin	55.09	46.24 to 63.94	Yes	***	0.0001
romidepsin(2nM) vs. ixazomib+romidepsin	20.37	9.151 to 31.58	Yes	*	0.0142
ONC-7003					
carbo+taxol vs. ixazomib(50nM)	-25.78	-52.34 to 0.7757	No	ns	0.0537
carbo+taxol vs. romidepsin(2nM)	32.26	20.30 to 44.23	Yes	**	0.0015
carbo+taxol vs. ixazomib+romidepsin	44.1	14.23 to 73.97	Yes	*	0.0198
ixazomib(50nM) vs. romidepsin(2nM)	58.05	32.63 to 83.46	Yes	**	0.0053
ixazomib(50nM) vs. ixazomib+romidepsin	69.89	41.84 to 97.93	Yes	**	0.0019
romidepsin(2nM) vs. ixazomib+romidepsin	11.84	-16.72 to 40.40	No	ns	0.3279
ONC-6163					
carbo+taxol vs. ixazomib(50nM)	37.19	-25.47 to 99.85	No	ns	0.1734
carbo+taxol vs. romidepsin(2nM)	41.8	12.55 to 71.05	Yes	*	0.0168
carbo+taxol vs. ixazomib+romidepsin	76.86	38.47 to 115.3	Yes	*	0.013
ixazomib(50nM) vs. romidepsin(2nM)	4.609	-64.24 to 73.46	No	ns	0.9766
ixazomib(50nM) vs. ixazomib+romidepsin	39.67	-39.24 to 118.6	No	ns	0.1756
romidepsin(2nM) vs. ixazomib+romidepsin	35.06	9.964 to 60.16	Yes	*	0.0262
NR3					
carbo+taxol vs. ixazomib(50nM)	-5.049	-27.12 to 17.02	No	ns	0.6655
carbo+taxol vs. romidepsin(2nM)	-3.588	-12.11 to 4.929	No	ns	0.4156
carbo+taxol vs. ixazomib+romidepsin	36.52	27.70 to 45.34	Yes	***	0.0003
ixazomib(50nM) vs. romidepsin(2nM)	1.461	-21.42 to 24.34	No	ns	0.9811
ixazomib(50nM) vs. ixazomib+romidepsin	41.57	19.21 to 63.94	Yes	*	0.0109
romidepsin(2nM) vs. ixazomib+romidepsin	40.11	31.93 to 48.30	Yes	***	0.0002
ONC-6562					
carbo+taxol vs. ixazomib(50nM)	-8.059	-26.60 to 10.48	No	ns	0.3911
carbo+taxol vs. romidepsin(2nM)	23.75	6.573 to 40.92	Yes	*	0.0168
carbo+taxol vs. ixazomib+romidepsin	48.19	30.94 to 65.45	Yes	**	0.005
ixazomib(50nM) vs. romidepsin(2nM)	31.81	12.76 to 50.86	Yes	**	0.0085
ixazomib(50nM) vs. ixazomib+romidepsin	56.25	34.31 to 78.19	Yes	**	0.0066
romidepsin(2nM) vs. ixazomib+romidepsin	24.44	5.050 to 43.84	Yes	*	0.0302
ONC-7052					
carbo+taxol vs. ixazomib(50nM)	71.47	48.11 to 94.82	Yes	**	0.0025
carbo+taxol vs. romidepsin(2nM)	32.93	9.527 to 56.32	Yes	*	0.0157
carbo+taxol vs. ixazomib+romidepsin	79.8	50.67 to 108.9	Yes	**	0.0071
ixazomib(50nM) vs. romidepsin(2nM)	-38.54	-59.70 to -17.38	Yes	**	0.0095
ixazomib(50nM) vs. ixazomib+romidepsin	8.336	-5.246 to 21.92	No	ns	0.1249
romidepsin(2nM) vs. ixazomib+romidepsin	46.88	20.04 to 73.71	Yes	*	0.0169

ONC-7367					
carbo+taxol vs. ixazomib(50nM)	69.46	38.82 to 100.1	Yes	**	0.0099
carbo+taxol vs. romidepsin(2nM)	65.85	40.02 to 91.68	Yes	**	0.0047
carbo+taxol vs. ixazomib+romidepsin	73.34	42.16 to 104.5	Yes	**	0.0095
ixazomib(50nM) vs. romidepsin(2nM)	-3.613	-15.11 to 7.886	No	ns	0.4184
ixazomib(50nM) vs. ixazomib+romidepsin	3.88	1.007 to 6.752	Yes	*	0.0241
romidepsin(2nM) vs. ixazomib+romidepsin	7.492	-5.043 to 20.03	No	ns	0.1321
ONC-6007					
carbo+taxol vs. ixazomib(50nM)	9.394	-31.90 to 50.69	No	ns	0.5633
carbo+taxol vs. romidepsin(2nM)	25.05	19.47 to 30.63	Yes	***	0.0007
carbo+taxol vs. ixazomib+romidepsin	28.63	5.121 to 52.13	Yes	*	0.0326
ixazomib(50nM) vs. romidepsin(2nM)	15.65	-26.96 to 58.27	No	ns	0.2953
ixazomib(50nM) vs. ixazomib+romidepsin	19.23	-13.82 to 52.29	No	ns	0.1984
romidepsin(2nM) vs. ixazomib+romidepsin	3.58	-21.70 to 28.86	No	ns	0.7942
ONC-6140					
carbo+taxol vs. ixazomib(50nM)	-35.64	-55.23 to -16.05	Yes	**	0.0063
carbo+taxol vs. romidepsin(2nM)	28.33	6.926 to 49.73	Yes	*	0.0287
carbo+taxol vs. ixazomib+romidepsin	32.41	15.42 to 49.40	Yes	**	0.0078
ixazomib(50nM) vs. romidepsin(2nM)	63.97	39.58 to 88.37	Yes	**	0.0075
ixazomib(50nM) vs. ixazomib+romidepsin	68.05	48.31 to 87.80	Yes	**	0.0017
romidepsin(2nM) vs. ixazomib+romidepsin	4.084	-7.088 to 15.26	No	ns	0.3196
ONC-6191					
carbo+taxol vs. ixazomib(50nM)	32.4	23.20 to 41.60	Yes	**	0.0017
carbo+taxol vs. romidepsin(2nM)	35.5	26.27 to 44.73	Yes	***	0.0004
carbo+taxol vs. ixazomib+romidepsin	66.33	54.84 to 77.82	Yes	***	0.0003
ixazomib(50nM) vs. romidepsin(2nM)	3.102	-5.244 to 11.45	No	ns	0.4171
ixazomib(50nM) vs. ixazomib+romidepsin	33.93	28.54 to 39.33	Yes	****	<0.0001
romidepsin(2nM) vs. ixazomib+romidepsin	30.83	20.24 to 41.43	Yes	**	0.0063
PDX1					
carbo+taxol vs. ixazomib(50nM)	16.97	-23.14 to 57.07	No	ns	0.2617
carbo+taxol vs. romidepsin(2nM)	29.68	-10.74 to 70.11	No	ns	0.094
carbo+taxol vs. ixazomib+romidepsin	57.14	21.49 to 92.80	Yes	*	0.015
ixazomib(50nM) vs. romidepsin(2nM)	12.71	4.042 to 21.39	Yes	*	0.0136
ixazomib(50nM) vs. ixazomib+romidepsin	40.18	25.37 to 54.98	Yes	**	0.0026
romidepsin(2nM) vs. ixazomib+romidepsin	27.46	12.52 to 42.40	Yes	**	0.009
PDX2					

carbo+taxol vs. ixazomib(50nM)	13.04	-21.27 to 47.35	No	ns	0.4655
carbo+taxol vs. romidepsin(2nM)	36.51	6.655 to 66.36	Yes	*	0.0259
carbo+taxol vs. ixazomib+romidepsin	60.35	28.78 to 91.92	Yes	*	0.0136
ixazomib(50nM) vs. romidepsin(2nM)	23.47	-11.69 to 58.63	No	ns	0.1594
ixazomib(50nM) vs. ixazomib+romidepsin	47.31	3.002 to 91.62	Yes	*	0.044
romidepsin(2nM) vs. ixazomib+romidepsin	23.84	-13.48 to 61.16	No	ns	0.1174

PDX3

carbo+taxol vs. ixazomib(50nM)	-14.17	-36.51 to 8.169	No	ns	0.1592
carbo+taxol vs. romidepsin(2nM)	-3.044	-20.20 to 14.11	No	ns	0.8699
carbo+taxol vs. ixazomib+romidepsin	-4.967	-24.24 to 14.30	No	ns	0.6918
ixazomib(50nM) vs. romidepsin(2nM)	11.13	-11.08 to 33.33	No	ns	0.298
ixazomib(50nM) vs. ixazomib+romidepsin	9.202	-13.59 to 31.99	No	ns	0.446
romidepsin(2nM) vs. ixazomib+romidepsin	-1.923	-21.96 to 18.11	No	ns	0.9767

ONC-6099

carboplatin+paclitaxel vs. bortezomib	28.19	19.80 to 36.57	Yes	****	<0.0001
carboplatin+paclitaxel vs. belinostat	-51.62	-60.01 to -43.23	Yes	****	<0.0001
carboplatin+paclitaxel vs. bortezomib+belinostat	35.21	26.83 to 43.60	Yes	****	<0.0001
bortezomib vs. belinostat	-79.81	-88.19 to -71.42	Yes	****	<0.0001
bortezomib vs. bortezomib+belinostat	7.026	-1.359 to 15.41	No	ns	0.1336
belinostat vs. bortezomib+belinostat	86.83	78.45 to 95.22	Yes	****	<0.0001

ONC-6051

carboplatin+paclitaxel vs. bortezomib	43.64	35.26 to 52.03	Yes	****	<0.0001
carboplatin+paclitaxel vs. belinostat	-7.444	-15.83 to 0.9419	No	ns	0.1007
carboplatin+paclitaxel vs. bortezomib+belinostat	44.71	36.32 to 53.09	Yes	****	<0.0001
bortezomib vs. belinostat	-51.09	-59.47 to -42.70	Yes	****	<0.0001
bortezomib vs. bortezomib+belinostat	1.065	-7.320 to 9.451	No	ns	0.9874
belinostat vs. bortezomib+belinostat	52.15	43.77 to 60.54	Yes	****	<0.0001

ONC-6057

carboplatin+paclitaxel vs. bortezomib	45.21	36.82 to 53.59	Yes	****	<0.0001
carboplatin+paclitaxel vs. belinostat	-36.11	-44.50 to -27.73	Yes	****	<0.0001
carboplatin+paclitaxel vs. bortezomib+belinostat	51.7	43.31 to 60.09	Yes	****	<0.0001
bortezomib vs. belinostat	-81.32	-89.71 to -72.94	Yes	****	<0.0001
bortezomib vs. bortezomib+belinostat	6.492	-1.894 to 14.88	No	ns	0.1872

belinostat vs. bortezomib+belinostat	87.81	79.43 to 96.20	Yes	****	<0.0001
ONC-6069					
carboplatin+paclitaxel vs. bortezomib	24	15.62 to 32.39	Yes	****	<0.0001
carboplatin+paclitaxel vs. belinostat	-12.29	-20.67 to -3.902	Yes	**	0.0012
carboplatin+paclitaxel vs. bortezomib+belinostat	42.19	33.81 to 50.58	Yes	****	<0.0001
bortezomib vs. belinostat	-36.29	-44.68 to -27.91	Yes	****	<0.0001
bortezomib vs. bortezomib+belinostat	18.19	9.805 to 26.58	Yes	****	<0.0001
belinostat vs. bortezomib+belinostat	54.48	46.10 to 62.87	Yes	****	<0.0001
ONC-6096					
carboplatin+paclitaxel vs. bortezomib	44.29	35.91 to 52.68	Yes	****	<0.0001
carboplatin+paclitaxel vs. belinostat	1.106	-7.280 to 9.491	No	ns	0.9859
carboplatin+paclitaxel vs. bortezomib+belinostat	42.19	33.81 to 50.58	Yes	****	<0.0001
bortezomib vs. belinostat	-43.19	-51.57 to -34.80	Yes	****	<0.0001
bortezomib vs. bortezomib+belinostat	-2.099	-10.48 to 6.286	No	ns	0.9144
belinostat vs. bortezomib+belinostat	41.09	32.70 to 49.47	Yes	****	<0.0001
ONC-6072					
carboplatin+paclitaxel vs. bortezomib	47.61	39.23 to 56.00	Yes	****	<0.0001
carboplatin+paclitaxel vs. belinostat	-19.96	-28.35 to -11.58	Yes	****	<0.0001
carboplatin+paclitaxel vs. bortezomib+belinostat	53.22	44.83 to 61.60	Yes	****	<0.0001
bortezomib vs. belinostat	-67.57	-75.96 to -59.19	Yes	****	<0.0001
bortezomib vs. bortezomib+belinostat	5.603	-2.782 to 13.99	No	ns	0.3067
belinostat vs. bortezomib+belinostat	73.18	64.79 to 81.56	Yes	****	<0.0001
ONC-6092					
carboplatin+paclitaxel vs. bortezomib	46.52	38.14 to 54.91	Yes	****	<0.0001
carboplatin+paclitaxel vs. belinostat	13.25	4.863 to 21.63	Yes	***	0.0004
carboplatin+paclitaxel vs. bortezomib+belinostat	63.79	55.40 to 72.17	Yes	****	<0.0001
bortezomib vs. belinostat	-33.27	-41.66 to -24.89	Yes	****	<0.0001
bortezomib vs. bortezomib+belinostat	17.27	8.880 to 25.65	Yes	****	<0.0001
belinostat vs. bortezomib+belinostat	50.54	42.15 to 58.92	Yes	****	<0.0001
ONC-6191					
carboplatin+paclitaxel vs. bortezomib	-4.936	-13.32 to 3.450	No	ns	0.4202
carboplatin+paclitaxel vs. belinostat	5.527	-2.859 to 13.91	No	ns	0.3188

carboplatin+paclitaxel vs. bortezomib+belinostat	66.55	58.17 to 74.94	Yes	****	<0.0001
bortezomib vs. belinostat	10.46	2.077 to 18.85	Yes	**	0.0081
bortezomib vs. bortezomib+belinostat	71.49	63.10 to 79.87	Yes	****	<0.0001
belinostat vs. bortezomib+belinostat	61.02	52.64 to 69.41	Yes	****	<0.0001
ONC-6134					
carboplatin+paclitaxel vs. bortezomib	15.03	6.640 to 23.41	Yes	****	<0.0001
carboplatin+paclitaxel vs. belinostat	14.12	5.738 to 22.51	Yes	***	0.0001
carboplatin+paclitaxel vs. bortezomib+belinostat	59.08	50.69 to 67.46	Yes	****	<0.0001
bortezomib vs. belinostat	-0.9018	-9.287 to 7.484	No	ns	0.9923
bortezomib vs. bortezomib+belinostat	44.05	35.67 to 52.44	Yes	****	<0.0001
belinostat vs. bortezomib+belinostat	44.96	36.57 to 53.34	Yes	****	<0.0001
ONC-6071					
carboplatin+paclitaxel vs. bortezomib	68.75	60.36 to 77.13	Yes	****	<0.0001
carboplatin+paclitaxel vs. belinostat	4.013	-4.373 to 12.40	No	ns	0.5978
carboplatin+paclitaxel vs. bortezomib+belinostat	83.11	74.73 to 91.50	Yes	****	<0.0001
bortezomib vs. belinostat	-64.73	-73.12 to -56.35	Yes	****	<0.0001
bortezomib vs. bortezomib+belinostat	14.36	5.979 to 22.75	Yes	***	0.0001
belinostat vs. bortezomib+belinostat	79.1	70.71 to 87.48	Yes	****	<0.0001
ONC-6061					
carboplatin+paclitaxel vs. bortezomib	61.7	53.32 to 70.09	Yes	****	<0.0001
carboplatin+paclitaxel vs. belinostat	3.643	-4.742 to 12.03	No	ns	0.6699
carboplatin+paclitaxel vs. bortezomib+belinostat	80.15	71.76 to 88.53	Yes	****	<0.0001
bortezomib vs. belinostat	-58.06	-66.45 to -49.68	Yes	****	<0.0001
bortezomib vs. bortezomib+belinostat	18.44	10.06 to 26.83	Yes	****	<0.0001
belinostat vs. bortezomib+belinostat	76.5	68.12 to 84.89	Yes	****	<0.0001
ONC-6173					
carboplatin+paclitaxel vs. bortezomib	64.91	56.52 to 73.29	Yes	****	<0.0001
carboplatin+paclitaxel vs. belinostat	32.67	24.29 to 41.06	Yes	****	<0.0001
carboplatin+paclitaxel vs. bortezomib+belinostat	83.93	75.54 to 92.31	Yes	****	<0.0001
bortezomib vs. belinostat	-32.24	-40.62 to -23.85	Yes	****	<0.0001
bortezomib vs. bortezomib+belinostat	19.02	10.63 to 27.40	Yes	****	<0.0001
belinostat vs. bortezomib+belinostat	51.25	42.87 to 59.64	Yes	****	<0.0001
ONC-5942					

carboplatin+paclitaxel vs. bortezomib	59.89	51.50 to 68.27	Yes	****	<0.0001
carboplatin+paclitaxel vs. belinostat	24.5	16.11 to 32.88	Yes	****	<0.0001
carboplatin+paclitaxel vs. bortezomib+belinostat	76.05	67.66 to 84.43	Yes	****	<0.0001
bortezomib vs. belinostat	-35.39	-43.78 to -27.00	Yes	****	<0.0001
bortezomib vs. bortezomib+belinostat	16.16	7.774 to 24.54	Yes	****	<0.0001
belinostat vs. bortezomib+belinostat	51.55	43.16 to 59.93	Yes	****	<0.0001
ONC-6107					
carboplatin+paclitaxel vs. bortezomib	54.29	45.90 to 62.68	Yes	****	<0.0001
carboplatin+paclitaxel vs. belinostat	11.29	2.906 to 19.68	Yes	**	0.0035
carboplatin+paclitaxel vs. bortezomib+belinostat	95.03	86.64 to 103.4	Yes	****	<0.0001
bortezomib vs. belinostat	-43	-51.38 to -34.61	Yes	****	<0.0001
bortezomib vs. bortezomib+belinostat	40.74	32.35 to 49.12	Yes	****	<0.0001
belinostat vs. bortezomib+belinostat	83.73	75.35 to 92.12	Yes	****	<0.0001
